# Supplementary material for: Trigonometric gradient microstructures in additively manufactured single crystals enable strength-ductility synergy and programmable performance
Source: Nat Commun. 2025 Nov 11;16:9936. doi: 10.1038/s41467-025-64874-1 (PMC12606096; doi:10.1038/s41467-025-64874-1)
Supplement: Supplementary file 1 — Supplementary Information [file 41467_2025_64874_MOESM1_ESM.pdf]

Supplementary Materials for

Trigonometric Gradient Microstructures in Additively Manufactured Single Crystals Enable Strength-Ductility Synergy and Programmable Performance

Zixu Guo[1,#], Yang Li[2,#], Lei Fan[1], Shiwei Wu[1], Daijun Hu[1], Guochen Peng[1],

Feng Lin[2], Yong-Wei Zhang[3], Yilun Xu*[,3,4], Wentao Yan*[,1]

[1] Department of Mechanical Engineering, National University of Singapore, Singapore 117575, Singapore

[2] Department of Mechanical Engineering, Tsinghua University, Beijing, China

[3] Institute of High Performance Computing (IHPC), Agency for Science, Technology and Research (A*STAR), Singapore 138632, Singapore

[4] Department of Materials, Imperial College, London, SW7 2AZ, UK

* E-mail: yilun.xu@imperial.ac.uk (Yilun Xu); mpeyanw@nus.edu.sg (Wentao Yan)

# Equal contributions

This PDF includes:

Tables:

Table S1 Chemical compositions of AM SX alloy (in wt%)

Table S2 Processing parameters in EB-PBF

Table S3 Calibrated parameters in crystal plasticity model

Figures:

Fig. S1 Fabrication of AM SX alloy

Fig. S2 Comparison between trigonometric fitting and Gaussian fitting

Fig. S3 Statistical methods for precipitate area, width, length, and matrix channel width

Fig. S4 Distributions of solid-solution elements Co and Ti (in at. %)

Fig. S5 Thermal stability tests for TGMs

Fig. S6 Demonstration of residual stress/strain distributions

Fig. S7 Specimen preparation for dual-scale DIC

Fig. S8 Scalar GND density distribution calculated using DIC

Fig. S9 Distributions of TGMs as the inputs of CPFE simulations

Fig. S10 Simulated stress-strain responses under varying combinations of TGMs' intensities

Fig. S11 Low-cycle fatigue lifespan of AM SX with TGMs

Fig. S12 Simulation results at the early stage of deformation before the initiation of slip bands at room temperature (load *m* in Fig. S10 a)

Fig. S13 Simulation results at the late stage of deformation (load *n*' in Fig. S10 b) in the case of 980 °C

without slip band:

Fig. S14 Fractography analysis for the tested specimens at room temperature and 980°C

Fig. S15 CPFE parameter calibration results

Fig. S16 Benchmark against an as-cast Ni-based SX alloy

Fig. S17 Heat treatment at 1050 °C for 12 h (air cooled) to eliminate the gradients of microstructures

Fig. S18 Validation of the tunable performance by tailoring the TGMs using heat treatment at 1050 °C

Fig. S19 Variations in strength and elongation along the trajectory of heat treatment in Fig.5 f and i

Fig. S20 Schematic diagram illustrating the general role of TGMs' impacts on the mechanical properties of AM SXs:

Texts:

S1. Details of crystal plasticity modeling

Table S1 Chemical compositions of AM SX alloy (in wt%)

Ti	Al	Co	Cr	C
3.39	3.61	8.57	15.92	0.11
Nb	Ta	Mo	Si	B
0.93	1.85	1.86	0.038	0.0077
Fe	Cr	W	Mn	Ni
0.16	15.92	2.75	0.013	Balance

Table S2 Processing parameters in EB-PBF

Gun accelerating voltage (kV)	Line offset (mm)	Layer thickness (μm)	Beam power (W)	Support
60	0.1	70	300-1800	None
Area energy density (J/mm^2)	Scanning speed (m/s)	Focus offset (mA)	Preheating temperature (°C)	Substrate
6	0.5, 0.6, 0.7	12-20	1050	Stainless steel

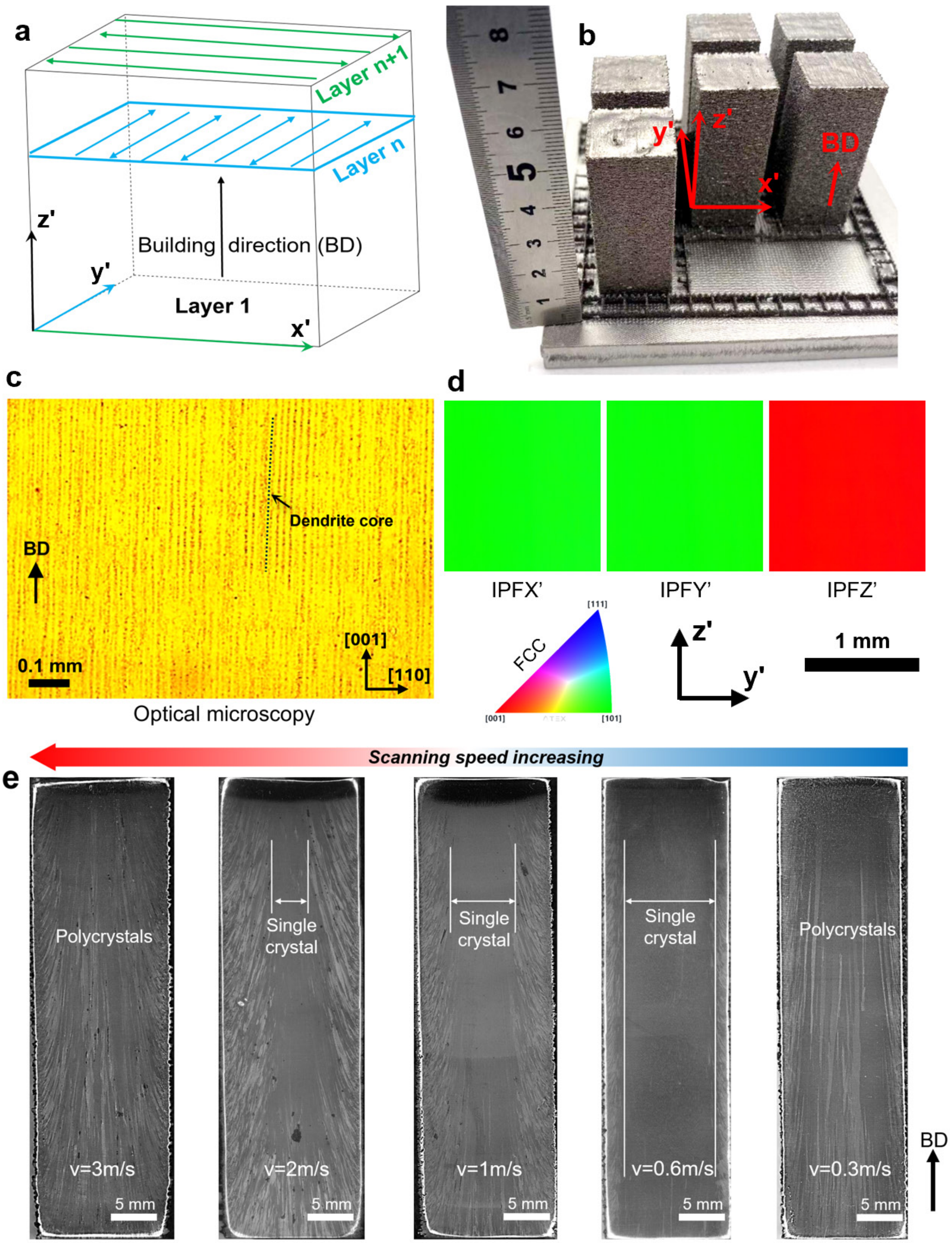

Fig. S1 Fabrication of AM SX alloy: (a) Scanning strategy used in the EB-PBF technique. **(b)** As-printed SX parts. **(c)** Optical microscopy image exhibits periodic distributions of dendrite cores and inter-dendrites at a large scale, with an average dendrite spacing of 15 μm. **(d)** Inverse pole figures (IPFs) within the gauge length of specimens, demonstrating that the printed SX alloy is grain boundary-free and crack-free on a large scale. **(e)** Selection of printing parameter[1], indicating that the largest SX can be obtained at a scanning speed of approximately 0.6 m/s.

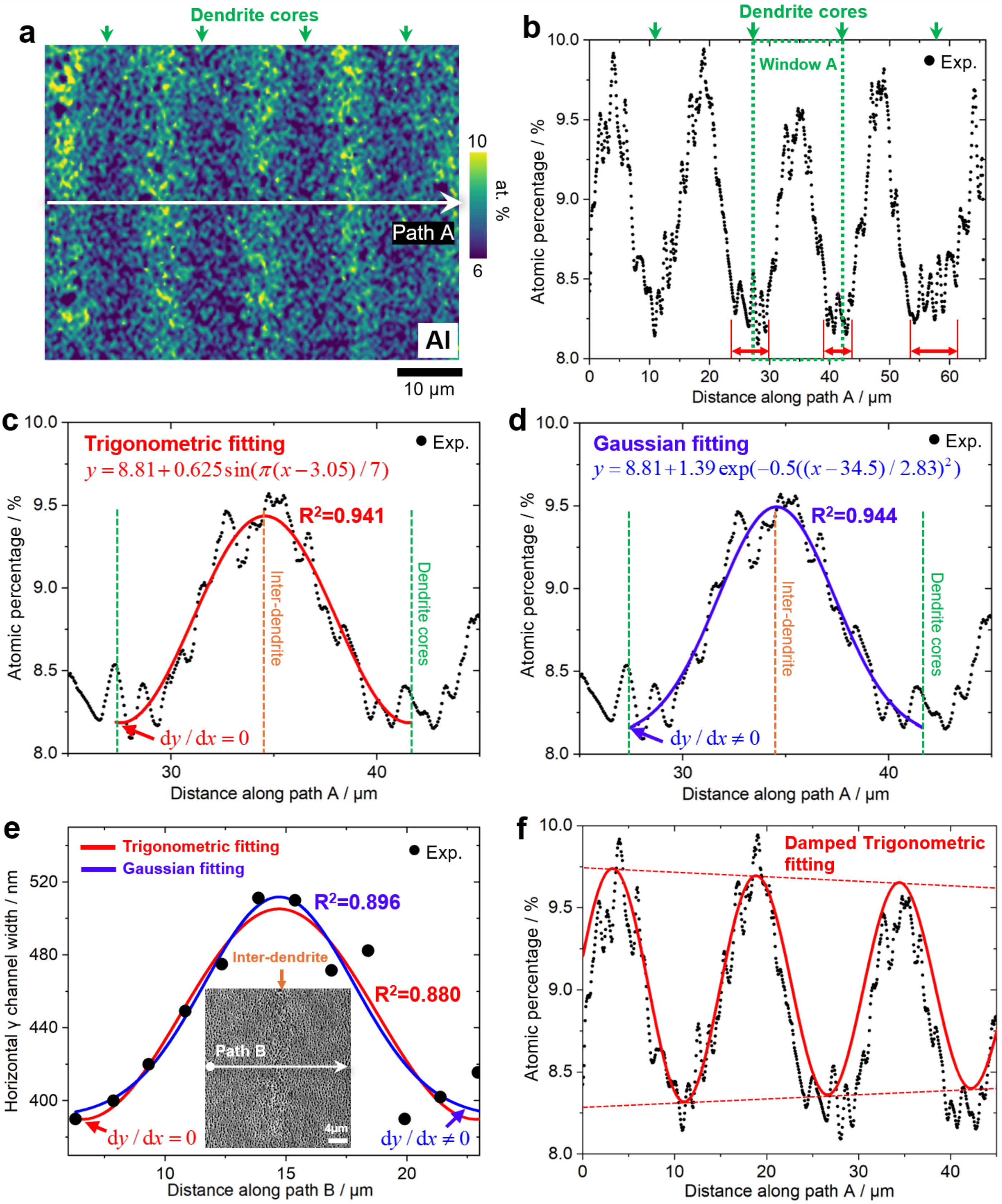

Fig. S2 Comparison between trigonometric fitting and Gaussian fitting: (a) Al distribution (in at. %) characterized using EPMA, which is employed as an example to test different fitting functions. **(b)** Distribution of Al atomic percentage along path A marked in **a**, in which each scatter is determined as the average within the height of the EPMA domain. **(c-d)** Fitting the experimental Al distribution within window A using trigonometric and Gaussian functions, respectively. **(e)** Distribution of γ matrix channel width surrounding an inter-dendrite (statistics approach detailed in **Fig. S3**), fitted using trigonometric and Gaussian functions. The correlation coefficients R^2 indicate that both functions exhibit comparable accuracy, similarly showing good agreement with the

statistical results in element distribution and γ/γ' morphology. However, since the derivative of a Gaussian function is nonzero everywhere, the Gaussian function fails to capture the boundary condition of $\mathrm{d}y/\mathrm{d}x \approx 0$ at dendrite cores, where the plateaus of gradient microstructure intensities with $\mathrm{d}y/\mathrm{d}x \approx 0$ (marked as the ranges of red double arrows in **b**) consistently exist in experiments. This arises from the inherent symmetry of gradient microstructures with respect to dendrite cores formed during the solidification process[2]. In addition, the trigonometric function is capable of representing the gradient microstructures distributed along multiple dendrites through a unified and concise equation, while the non-periodic Gaussian or polynomial functions fail to achieve this. Hence, the trigonometric function facilitates future investigations into the variations in the dendrite spacing and intensities of gradient microstructures among multiple dendrites. **(f)** An application case of damped trigonometric function[3] on multiple dendrites with varying peak height. Notably, since the element segregation shows a strong correlation with initial dislocations[4,5] and formation of γ' phases[6,7] governed by local chemical compositions, the above trends could be similarly applicable to other types of gradient microstructures around dendrites, since the plateaus with $\mathrm{d}y/\mathrm{d}x \approx 0$ also can be observed at dendrite cores for the precipitate area in **Fig 1 c3**.

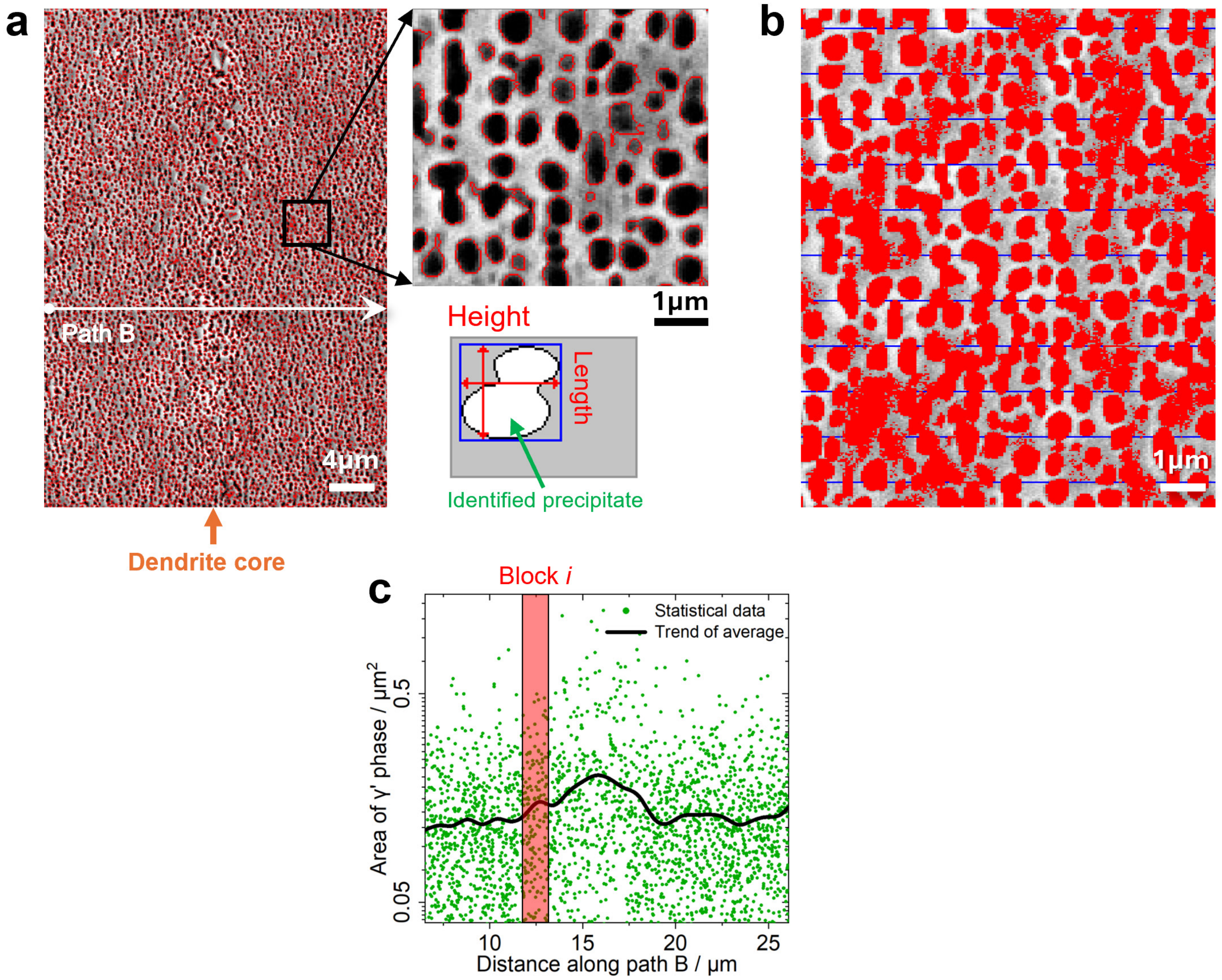

Fig. S3 Statistical methods for precipitate area, width, length, and matrix channel width: (a) Statistics on precipitate dimension and area using ImageJ-Pro Plus software[8], in which the central coordinates, height, length, and area for each precipitate can be accurately identified and extracted for further analysis. **(b)** Statistics on the horizontal width of γ matrix channel based on the chord length method for the dual phases[9], which is implemented based on our previous work[10]. The short blue lines represent the identified horizontal width of the matrix channel. **(c)** A statistical approach to capture the trend of variation in precipitate dimension and area along path B. For each γ' identified, the statistical results on precipitate area and coordinate are plotted in this figure as scatters. Then, path B is divided into 30 blocks, and statistical averaging is performed within each block. The resulting block-wise averages are then connected to form the 'trend of average' curve.

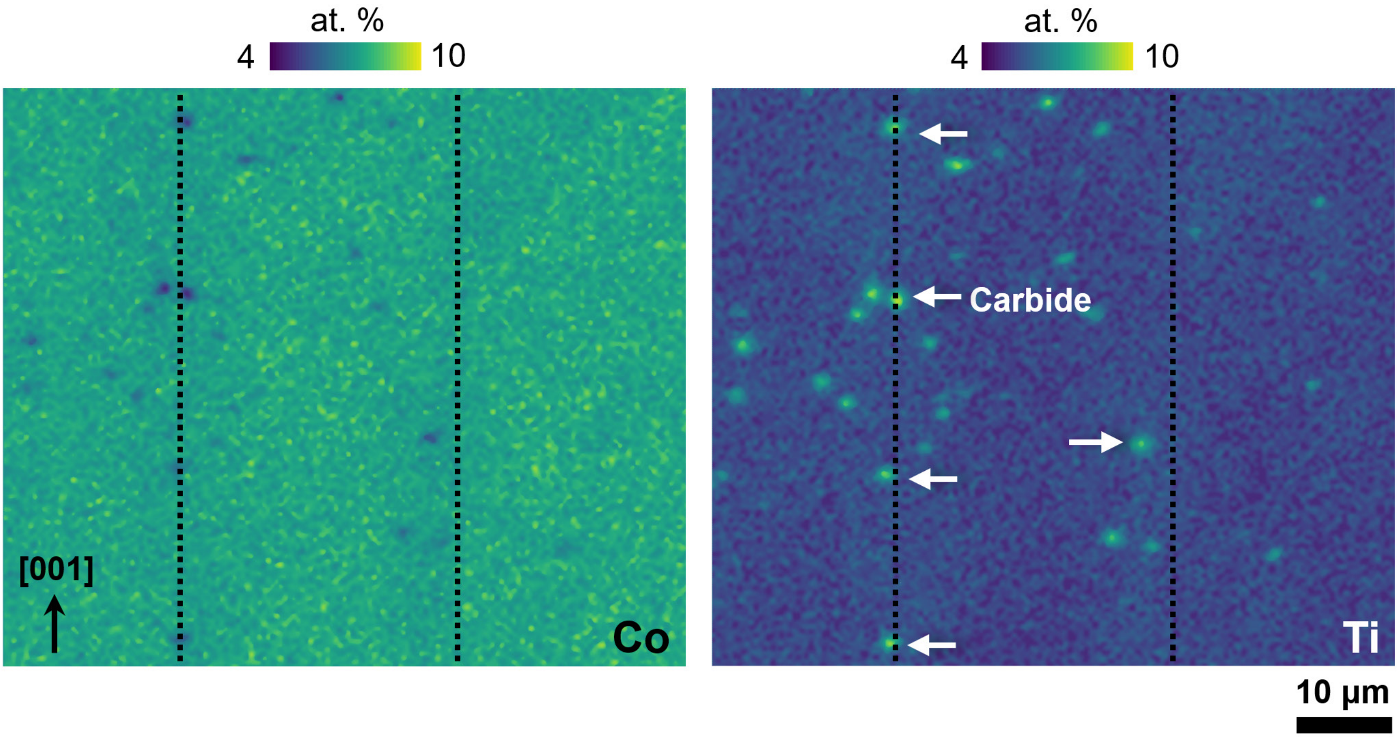

Fig. S4 Distributions of solid-solution elements Co and Ti (in at. %): The carbides distributed at inter-dendrites[11] are used to identify the locations of dendrite cores and inter-dendrites. Apart from Cr, Al, Ti, and Co, the other solid-solution elements exhibit negligible gradient distributions.

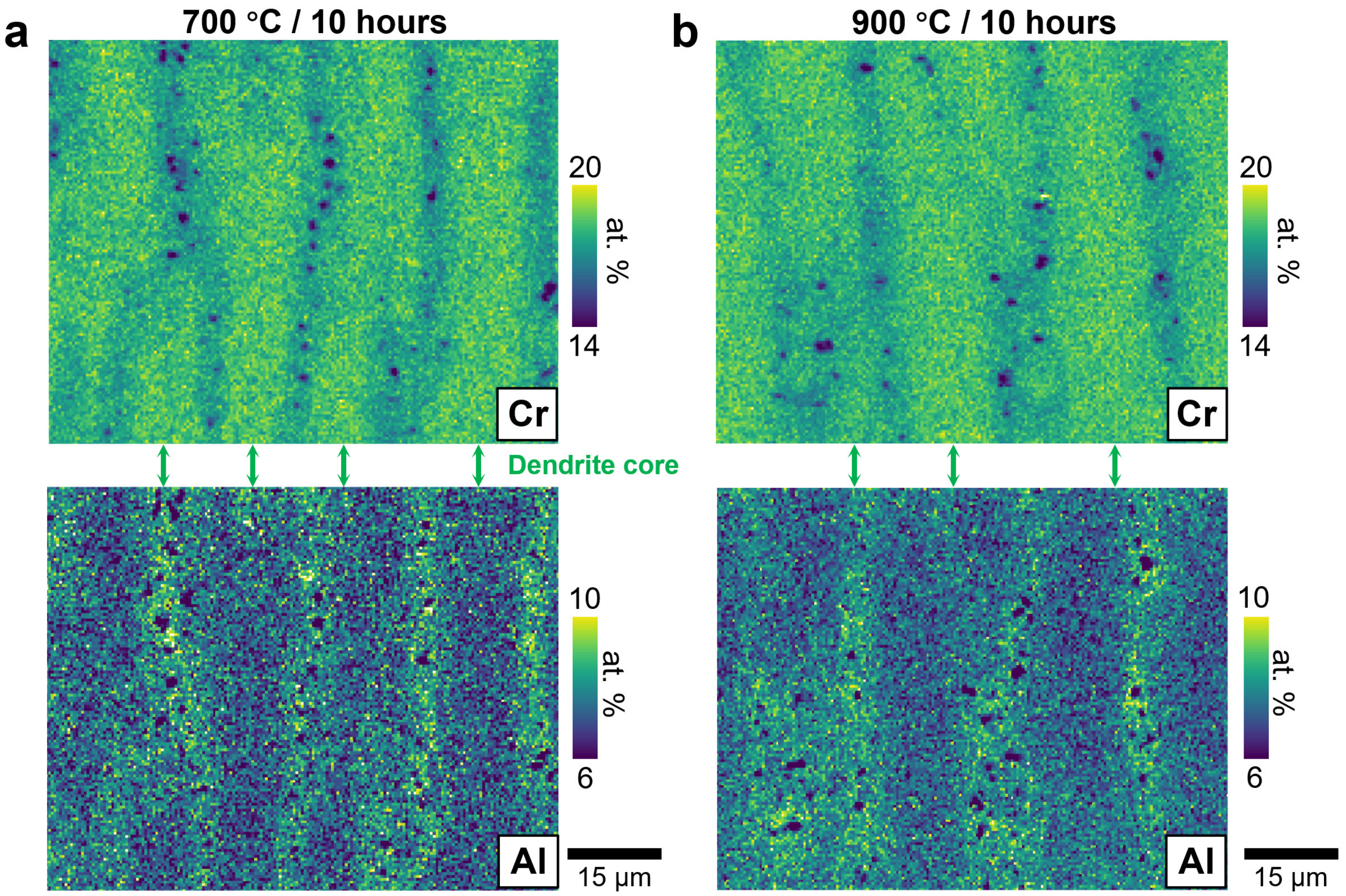

Fig. S5 Thermal stability test results for TGMs in AM SXs: (a)-(b) Distributions of solid-solution elements Cr and Al after heat exposure for 10 hours under 700 °C and 900 °C, respectively. By comparing with the solute segregations in the as-printed state (**Fig. 1 a** and **c**), the 700 °C and 900 °C heat exposure has minimal effects on the degree of segregation. The high temperatures ranging from 700 °C and 900 °C are insufficient to drive the diffusion of segregated solutes, as the diffusion is a thermally activated process. Furthermore, the above heat exposure is also unlikely to affect the initial density-graded dislocations, since the solute segregation is highly correlated with initial localized dislocations in AM alloys[4]. Therefore, the TGMs formed during the AM process show promising thermal stability after heat exposure from 700 °C to 900 °C for 10 hours, which are typical in-service temperatures of Ni-based superalloys[12].

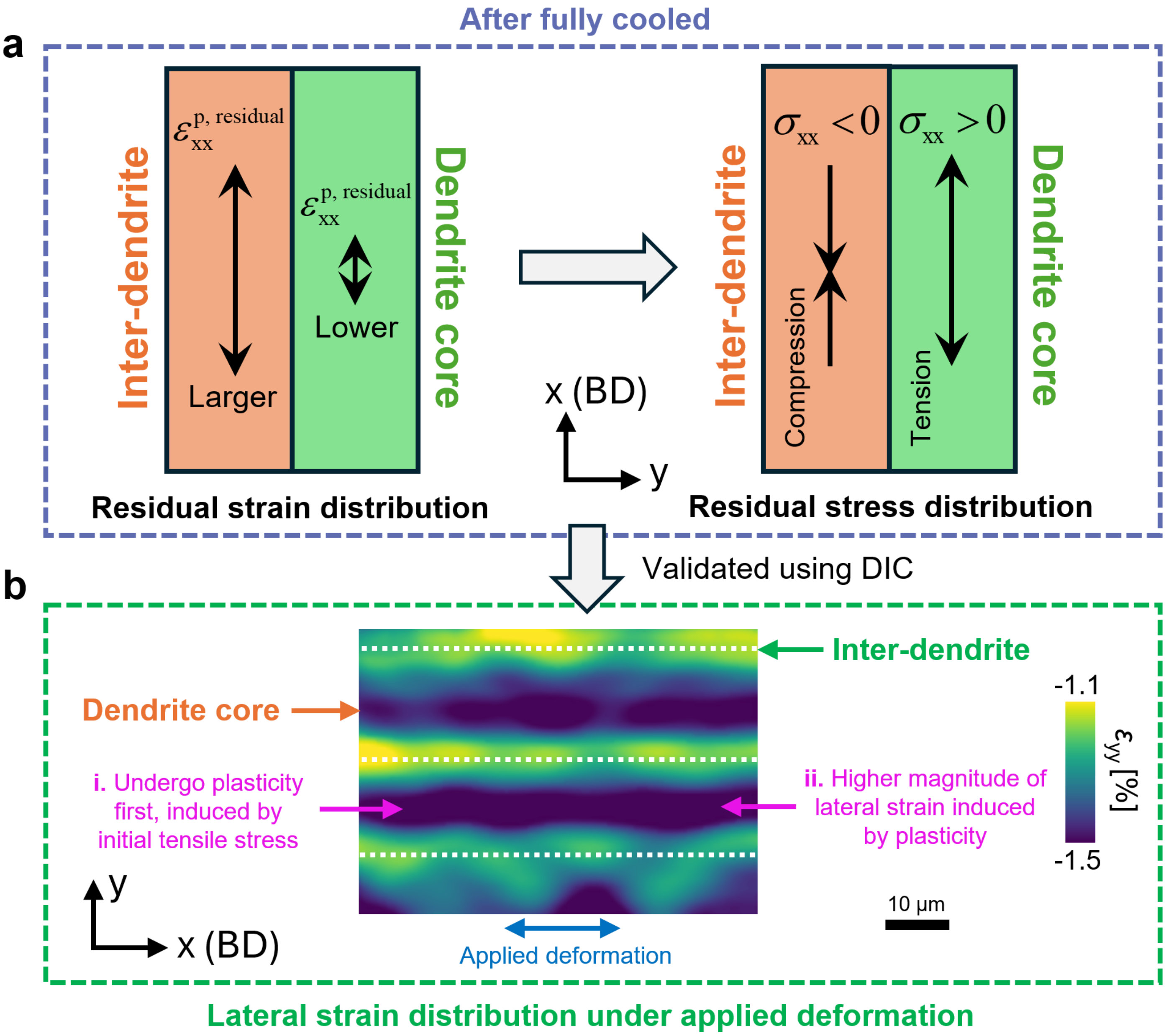

Fig. S6 Demonstration of residual stress/strain distributions: (a) Schematic diagram for the assumed directions of residual plastic strain and stress at the as-printed state. The inter-dendrite shows a higher residual tensile strain than the dendrite core. To maintain deformation compatibility, the inter-dendrite and dendrite core exhibit compressive and tensile residual stresses, respectively. **(b)** Experimental validation for the trend in **a**, based on the DIC analysis of lateral strain distribution at the early stage of deformation (**Fig 3 b2**). The periodic lateral strain pattern suggests that the dendrite cores undergo plasticity first, due to the residual tensile stress and weaker dislocation hardening at the dendrite cores. As a result, the dendrite cores exhibit a higher absolute value of ε_{yy}, which aligns well with the assumption in **a**. The revealed residual stress distribution across the dendrite core to inter-dendrite is consistent with the direct observation on residual stress for SX alloys using the FIB-DIC method[13].

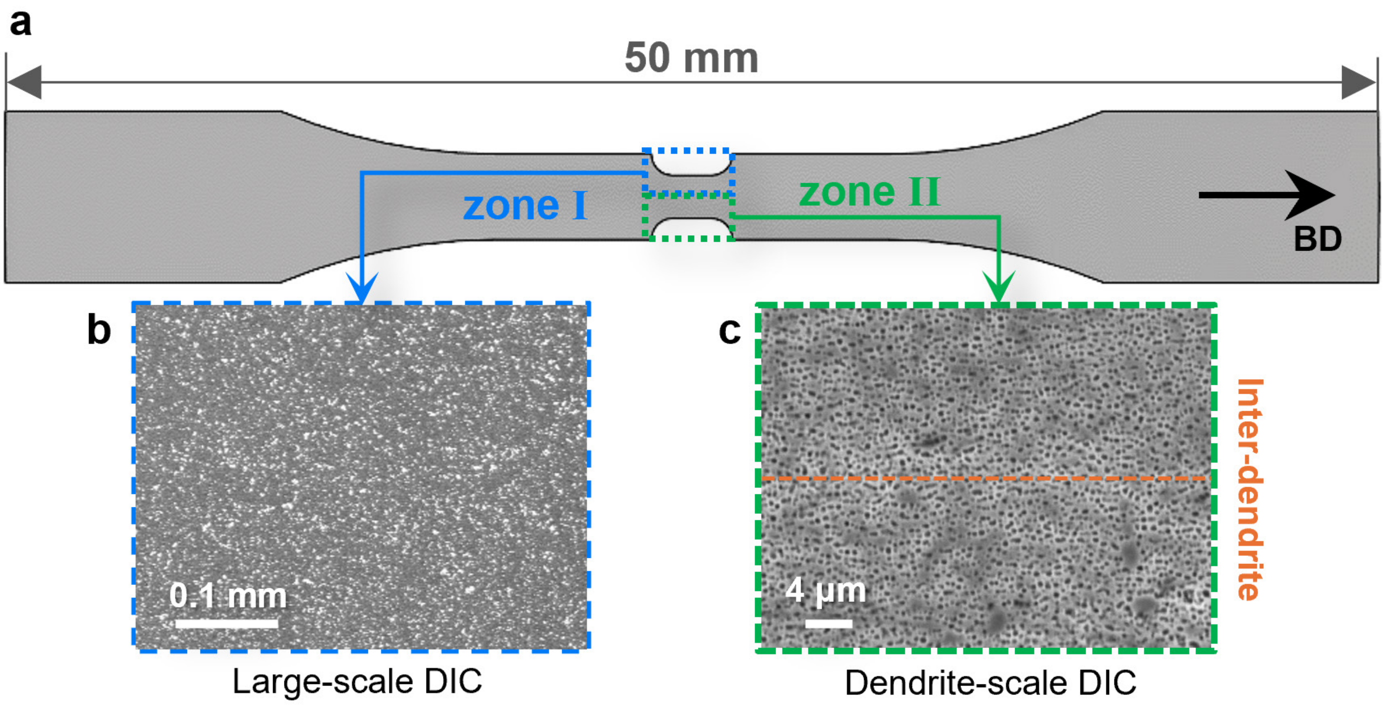

Fig. S7 Specimen preparation for dual-scale DIC: (a) Geometry of specimen for tensile test. **(b)** ZrO_2-based speckle pattern for millimeter-scale DIC. **(c)** γ/γ' microstructure-based speckle pattern for dendrite-scale DIC.

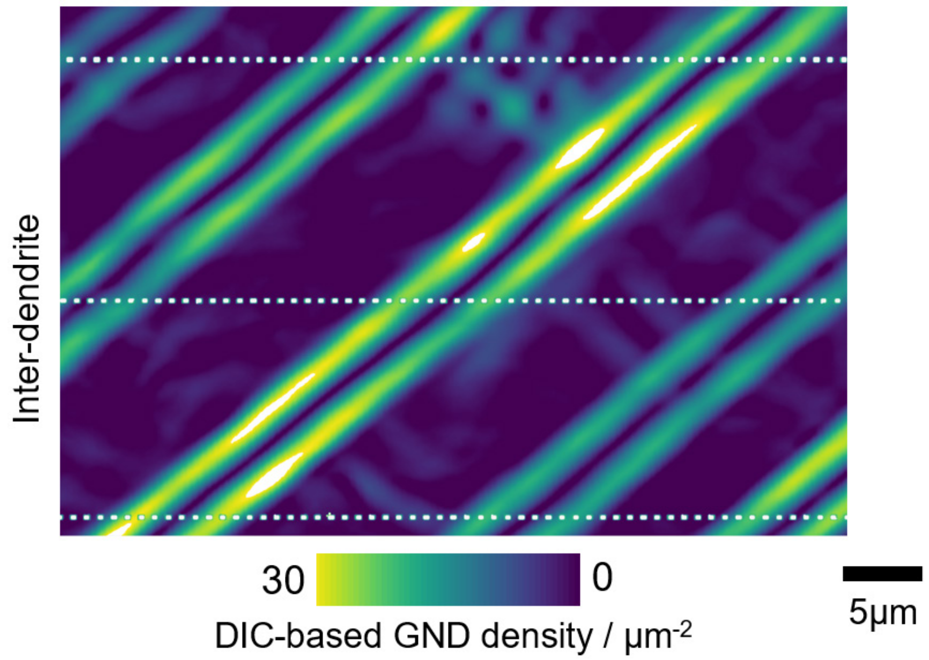

Fig. S8 Scalar GND density distribution calculated using DIC: DIC-based GND density is calculated based on the algorithm in literature[14]. The measurement is performed at 900 °C under 5% global strain, resembling the same loading condition of CPFE-based GND in **Fig. 4 h**. DIC-based GND density is primarily concentrated along the boundaries of each slip band, with the dendrite core exhibiting higher GND densities than inter-dendrites. Both spatial distribution and magnitude of the DIC-derived GND density closely match those obtained from CPFE simulations in **Fig. 4 h**, providing further validation of the simulated results.

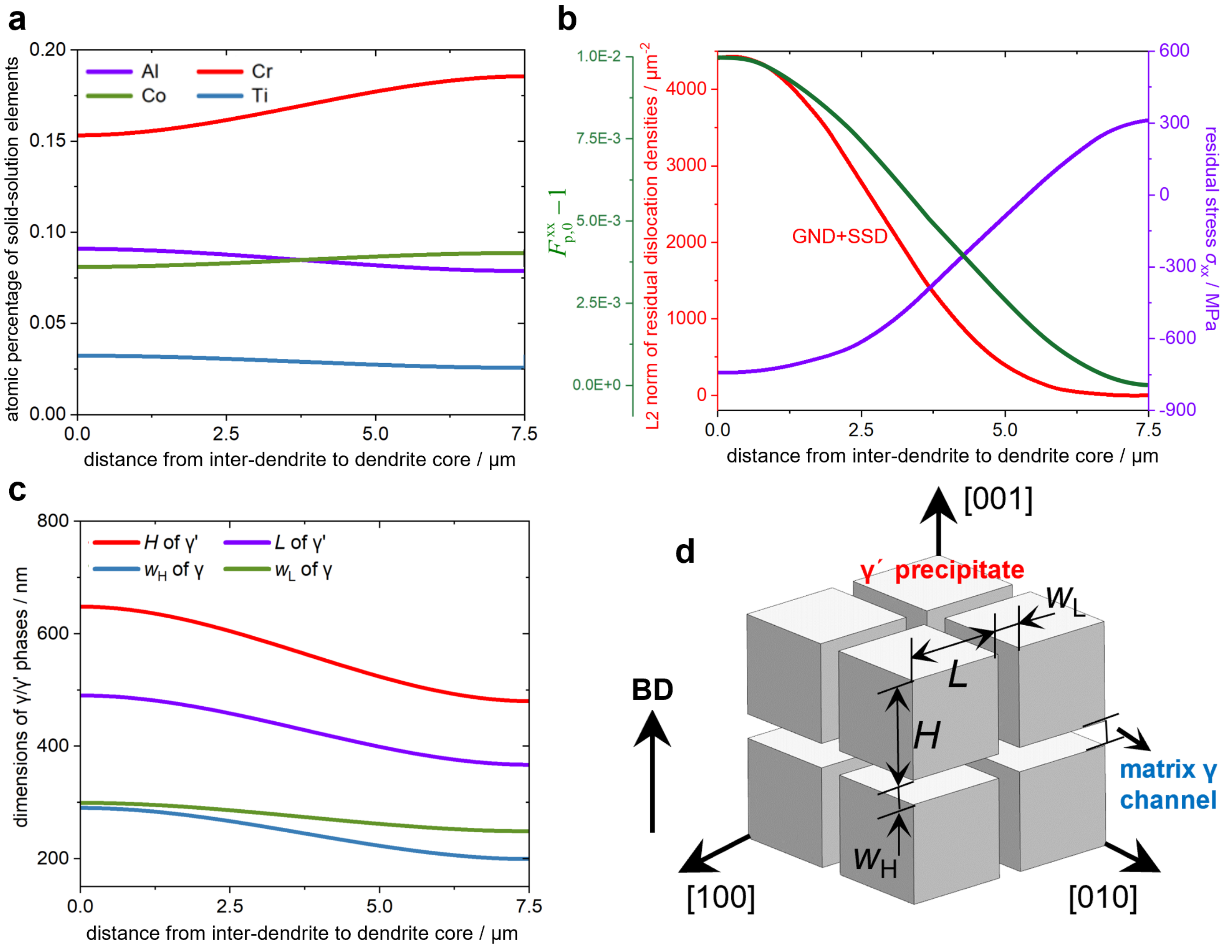

Fig. S9 Distributions of TGMs as the inputs of CPFE simulations: **(a)** Distributions of solid-solution elements along the path from inter-dendrite to dendrite core. **(b)** Distributions of residual deformation, initial dislocation densities, and residual stress σ_{xx} that accommodates the residual deformation. **(c)** Distributions for the dimensions of γ/γ' phases, which are determined by fitting the statistical results from SEM image processing. **(d)** Definitions of γ/γ' morphology parameters referred in **c**. The distributions in **a**, **c,** and the distribution of residual deformation in **b**, are quantified by the unified trigonometrical function developed in **Fig 1 a**.

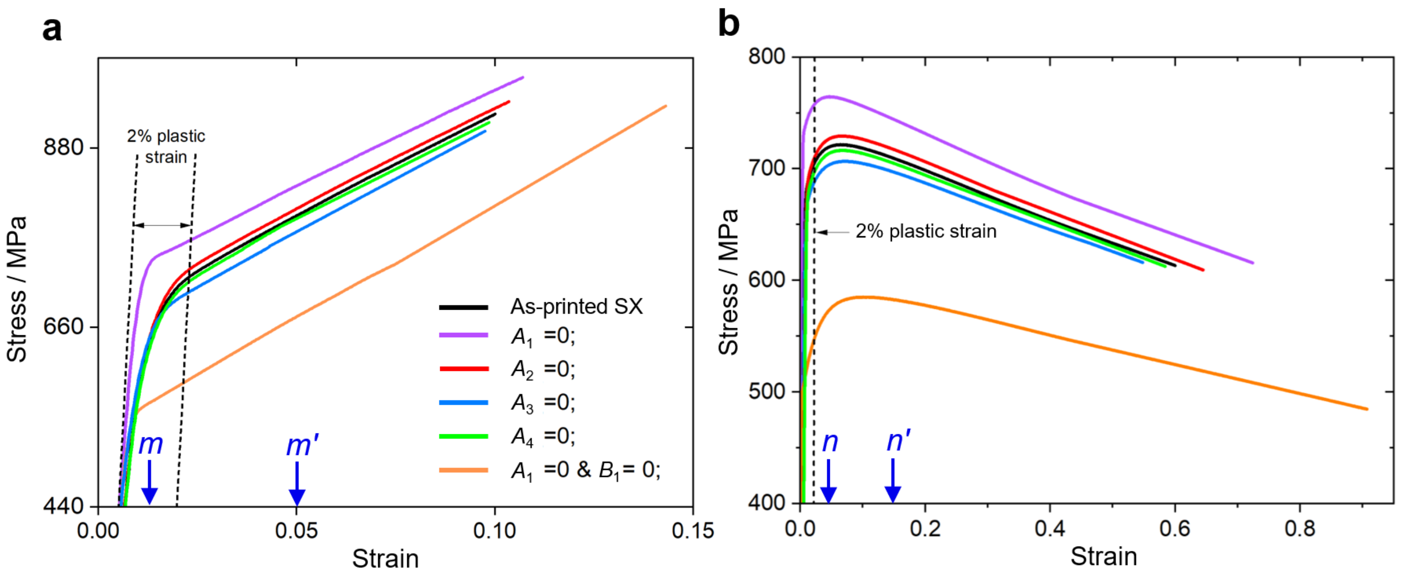

Fig. S10 Simulated stress-strain responses under varying combinations of TGMs' intensities: (a-b) Simulation results for the cases with and without slip band at RT and 980 °C, respectively. The stress corresponding to 2% plastic strain is used as a measure of strength, exhibiting significant differences in strength between the curves. The loads *m/n* and *m'/n'* correspond to the early and late stages of deformation mentioned in **Fig 4 a-b**, **e-f**, **Fig. S12**, and **Fig. S13**, respectively.

a

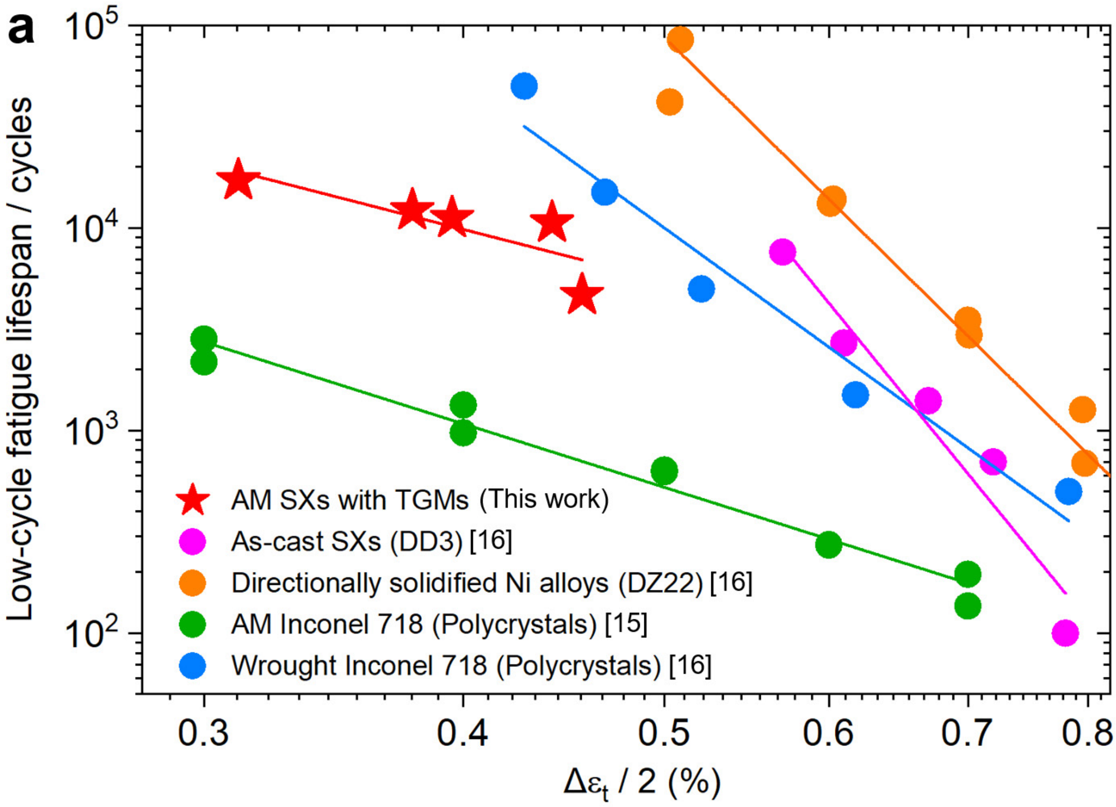

b

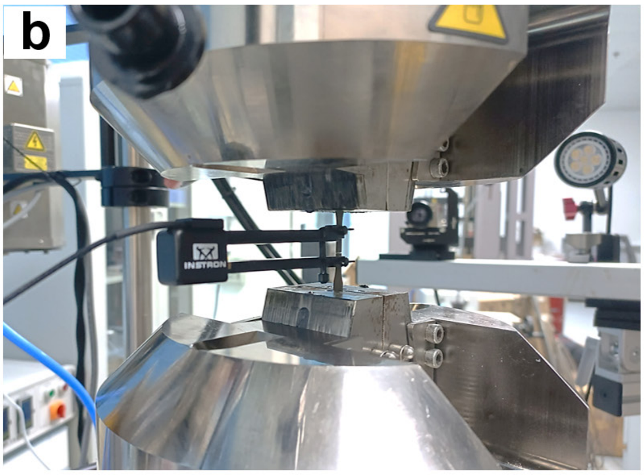

c

$\Delta\varepsilon_t/2$ (%)	LCF lifespan / cycles
0.31	1.72×10^4
0.4	1.22×10^4
0.44	1.12×10^4
0.38	1.06×10^4
0.46	4.67×10^3

Fig. S11 Low-cycle fatigue lifespans of AM SX with TGMs: (a) The fatigue lifespans of AM SX are compared with other Ni-based alloys with similar chemical compositions, including AM Inconel 718[15], as-cast SX/directionally solidified alloys[16], and wrought Inconel 718[16]. Compared with AM polycrystal Inconel 718, the AM SXs exhibit significantly improved fatigue performance. Furthermore, since fatigue life is more sensitive to AM-induced defects under low strain amplitude [17], both as-cast SX and wrought Inconel 718 demonstrate notably longer fatigue lifespans than AM SXs at low strain amplitudes. Nevertheless, when the half strain amplitude approximately exceeds 0.5%, the AM SX could achieve fatigue performance comparable to that of the as-cast SX and wrought Inconel 718. Therefore, the introduction of TGMs in the AM SX does not necessarily result in a pronounced degradation of fatigue performance. Although the TGMs enhance the interaction between dendrites and slip bands, the slip band formation is not unique to AM SXs with TGMs, and similarly plays a dominant role in the fatigue failure of other kinds of Ni-based alloys[18,19]. **(b)** The low-cycle fatigue tests for AM SX are conducted on a servo-hydraulic fatigue testing system (Instron 8801) using the strain-controlled mode, under a loading frequency of 2 Hz. **(c)** Raw data of fatigue lifespans and associated strain amplitudes from the LCF tests.

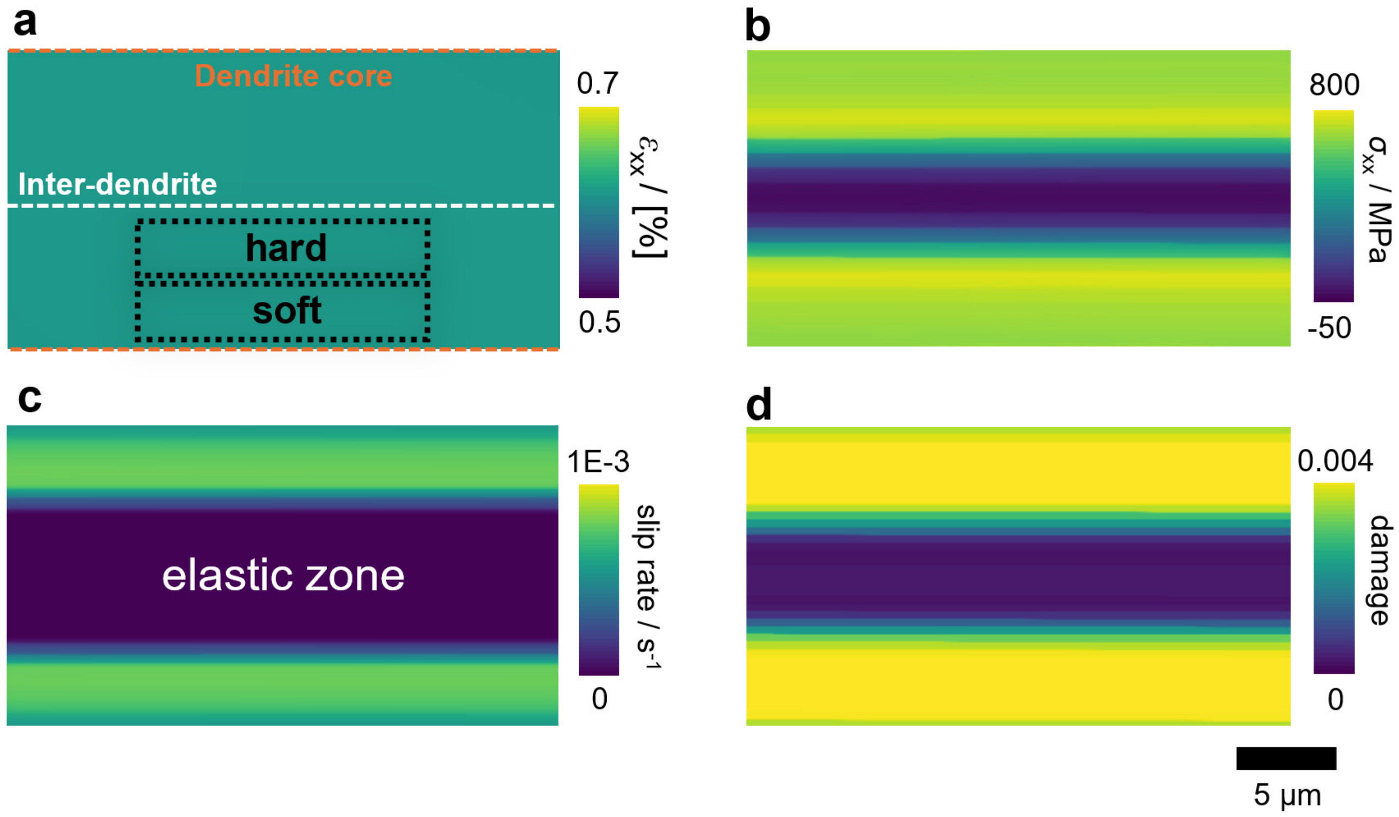

Fig. S12 Simulation results at the early stage of deformation before the initiation of slip bands at room temperature (load *m* in Fig. S10 a): (a) Strain distribution. **(b)** Stress distribution. **(c)** Distribution of slip rate (maximum among all slip systems). **(d)** Damage distribution. The whole process of deformation at room temperature is shown in **Supplementary Movie 1**.

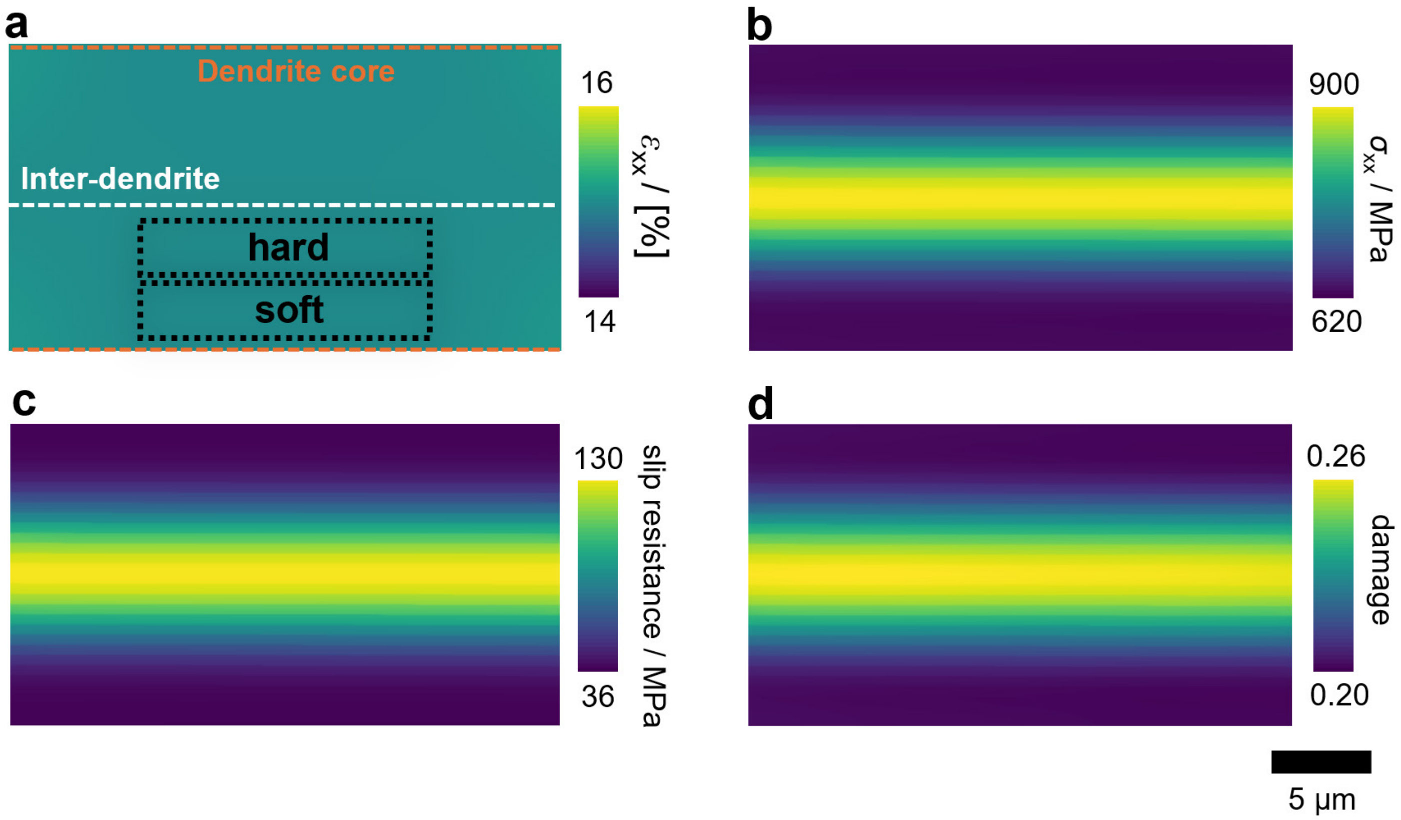

Fig. S13 Simulation results at the late stage of deformation (load *n'* in Fig. S10 b) in the case of 980 °C without slip band: (a) Strain distribution. **(b)** Stress distribution. **(c)** Distribution of slip resistance (maximum among all slip systems). **(d)** The damage is localized at the dendrite core, aligning well with the cracking events observed at dendrite cores (**Fig. S14 b**). The whole process of deformation at 980 °C is shown in **Supplementary Movie 2**.

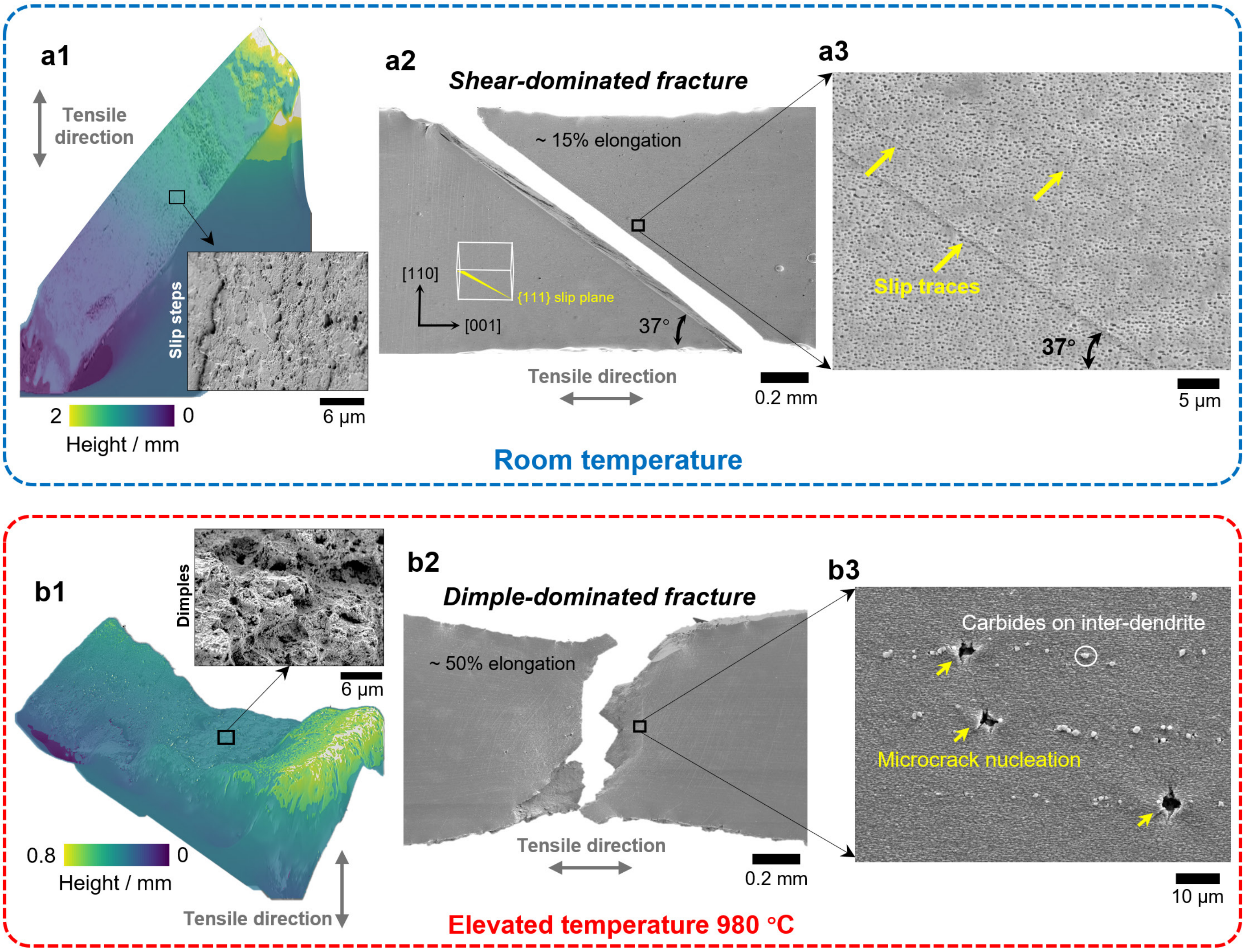

Fig. S14 Fractography analysis for the tested specimens at room temperature and 980°C: **(a1, b1)** Height maps for the fracture surface, using the 3D optical microscope (RX-2000, HiROX, Japan). **(a2, b2)** Macroscopic SEM images for the specimens fractured during the *in-situ* tensile tests. **(a3, b3)** Microstructures near the fracture surfaces. Based on the above observations, specimens with evident slip bands show shear-dominated fracture along the {111} slip planes, with dense slip traces observed near the fracture surface. This SEM observation aligns with the strain localization behavior within slip bands captured by DIC (**Fig. 3 a-d**). In these cases, failure is driven by slip and damage localization within the slip bands. On the other hand, specimens without slip bands display a more ductile fracture mode, characterized by a high density of dimples on the fracture surface. No slip traces are observed at 980 °C, as the applied stress is insufficient to activate γ′ phase shearing, which primarily governs the formation of slip traces[20]. In the case without slip traces, microcracks initiate around carbides at the inter-dendrite region due to the strain localization captured in DIC (**Fig. 3 i**). Therefore, the fractography analysis shows good agreement with DIC results. Moreover, these observed damage features can be well captured in our CPFE modeling: the simulated damage is localized within slip bands (**Fig. 4 c**) and inter-dendrites with carbides (**Fig. S11 d**), regarding the cases with and without slip bands, respectively. Hence, the simulated cracking sites with damage localization are in good agreement with the experimental observations at both room and elevated temperatures.

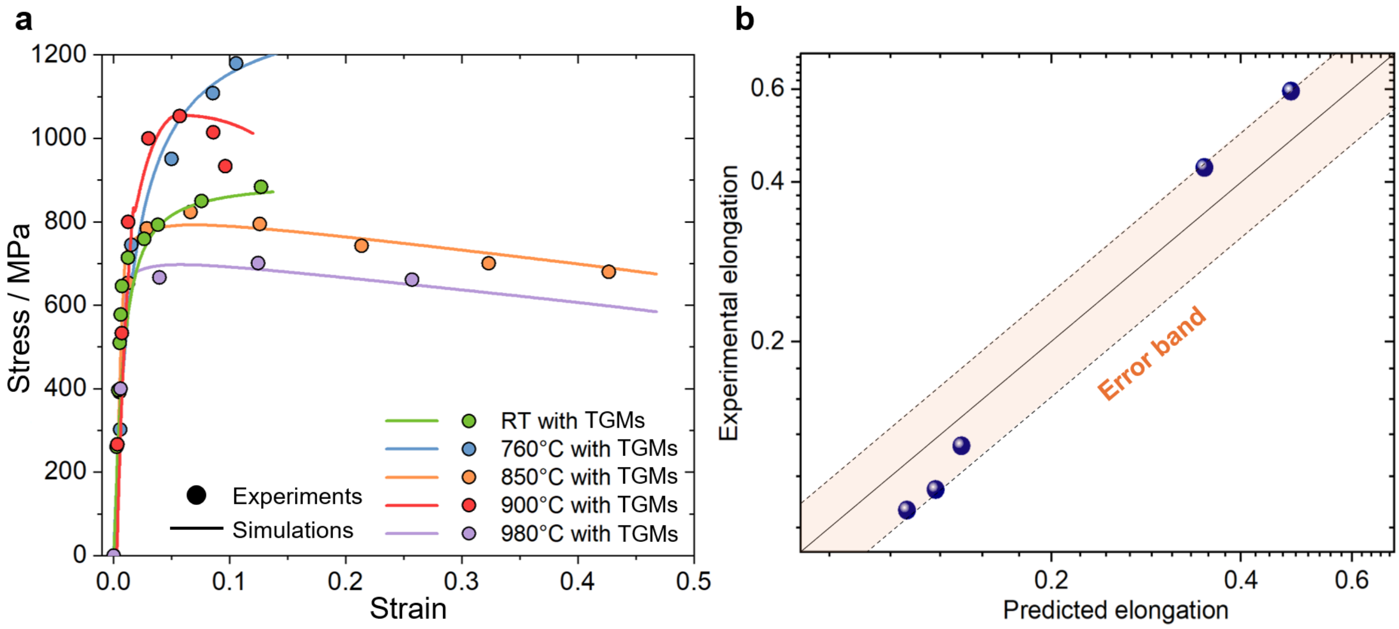

Fig. S15 CPFE parameter calibration results: (a) Comparison between simulated and experimental stress-strain curves. **(b)** Comparison between simulated and experimental elongation, indicating the relative errors fall within a range of -20%~25%, indicating that the CPFE model has acceptable accuracy in predicting elongations. Both **a** and **b** suggest considerable model accuracy.

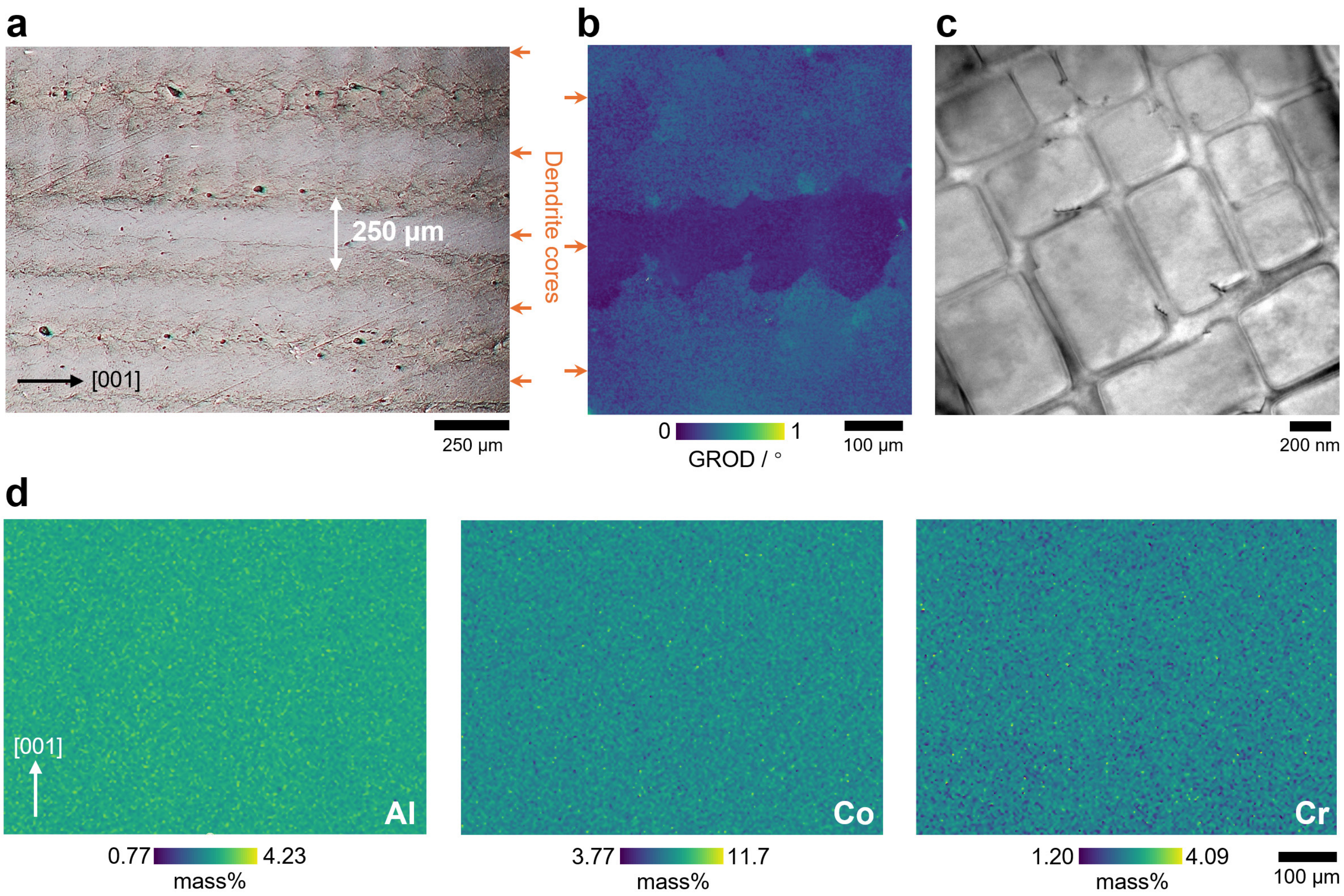

Fig. S16 Benchmark against an as-cast Ni-based SX alloy: (a) Morphology of dendrites under optical microscopy for an as-cast SX (trademark DD6, China), indicating that the columnar dendrite spacing of as-cast SX is nearly 250 μm, more than 15 times larger than that of AM SXs. **(b)** EBSD-based GROD map for as-cast SX exhibits negligible residual deformation distribution within each dendrite structure. **(c)** TEM observations on the inter-dendrite of the as-cast SX alloy, suggesting an absence of initial dislocations and associated density gradients. **(d)** EPMA results for solid-solution elements, showing minimal solute segregation across dendrite cores to inter-dendrites. Therefore, compared with AM SX alloys, as-cast SX alloys exhibit negligible initial dislocation densities and minimal solute segregation. As demonstrated in **Section 2.3**, since the TGM-induced simultaneous enhancement of strength and ductility primarily relies on the presence of initial density-graded dislocations, the proposed mechanism unique to AM SXs could not be applicable to as-cast SX alloys. Given that TGMs are introduced by the high cooling rates and large temperature gradients inherent to the AM process, the TGM mechanism is unique to AM alloys.

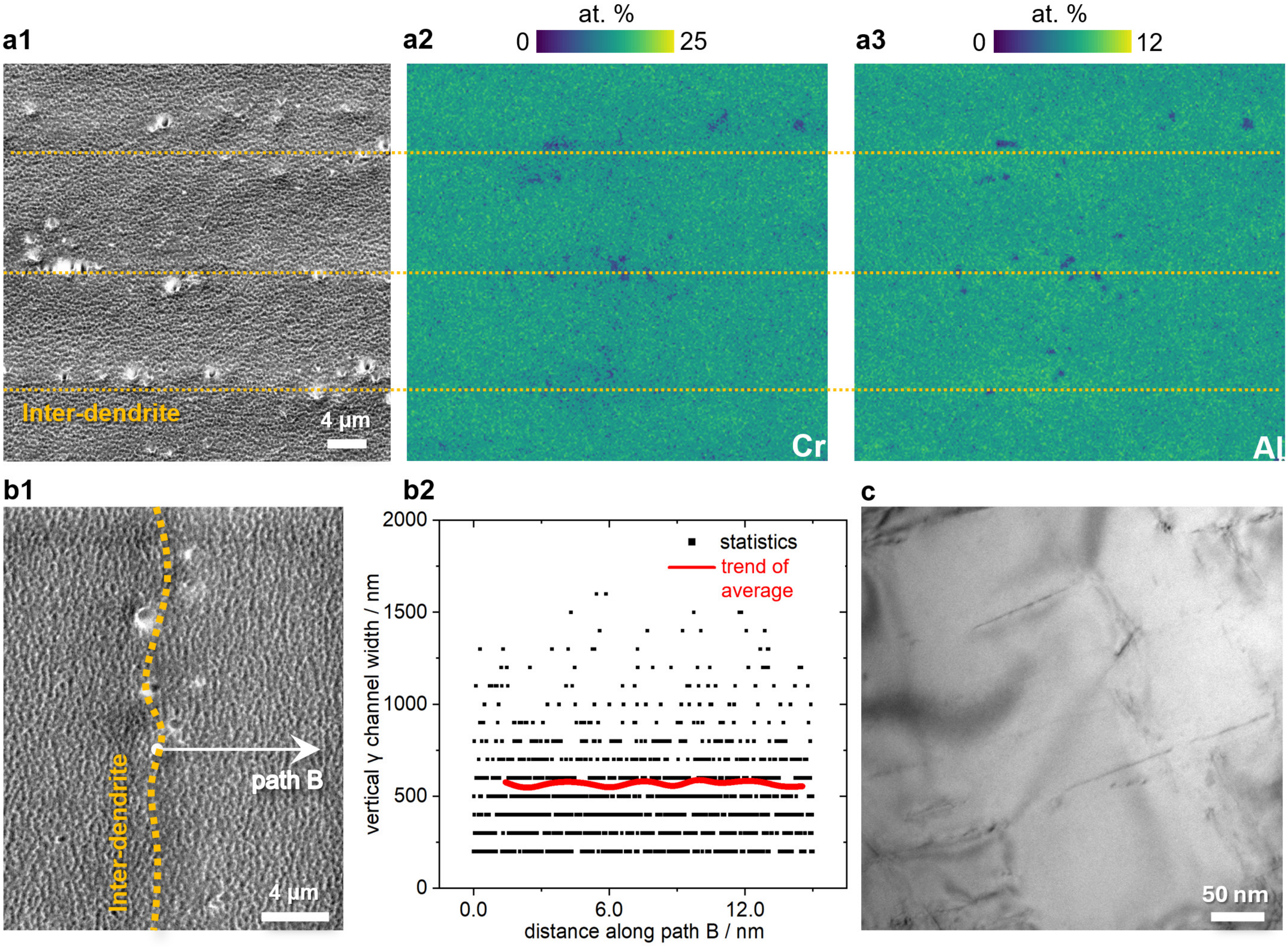

Fig. S17 Heat treatment at 1050 °C for 12 h (air cooled) to eliminate the gradients of microstructures: (a) Distributions of Cr and Al (in at. %). **(b)** γ/γ' morphology and statistical results of γ channel width. **(c)** TEM analysis for the region at inter-dendrite. **a-c** suggest that the gradients of microstructures formed in the AM process are fully eliminated after heat treatment at 1050 °C for 12 h.

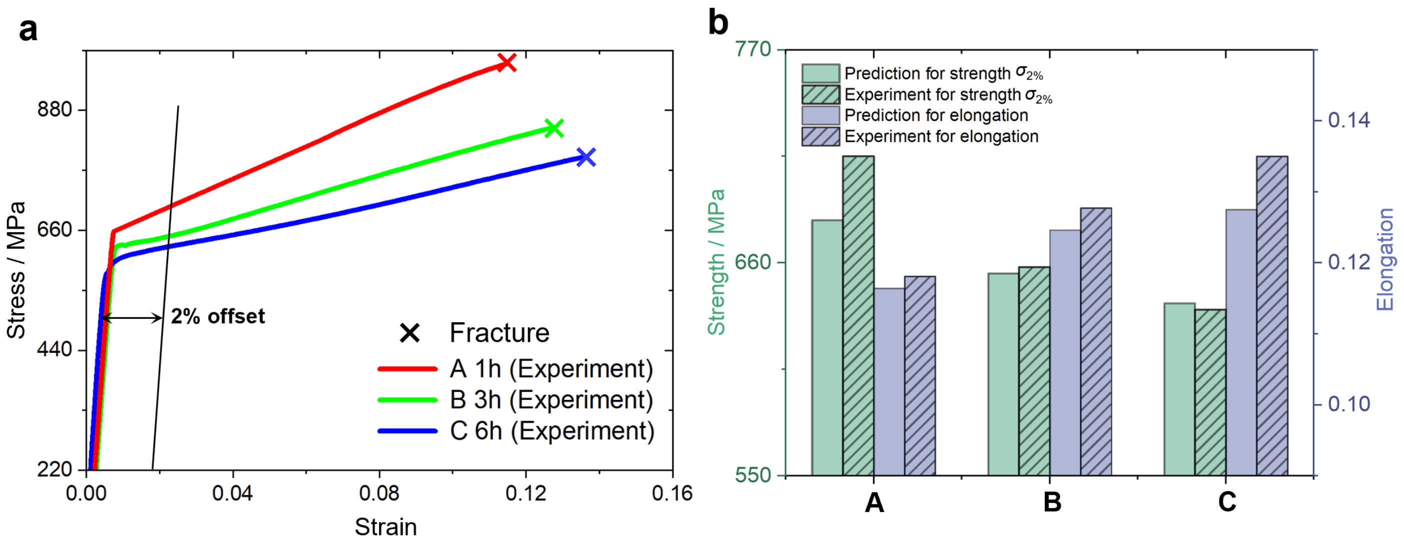

Fig. S18 Validation for the tunable performance by tailoring the TGMs using heat treatment at 1050 °C: (a) Experimental stress-strain curves for the A/B/C points marked in **Fig 5 d**. **(b)** Comparisons of strength and elongation between experiments and predictions for A/B/C points, indicating that the variation in strength and elongation at different heat treatment durations can be successfully predicted using the linear relationship between TGMs' intensities and mechanical properties.

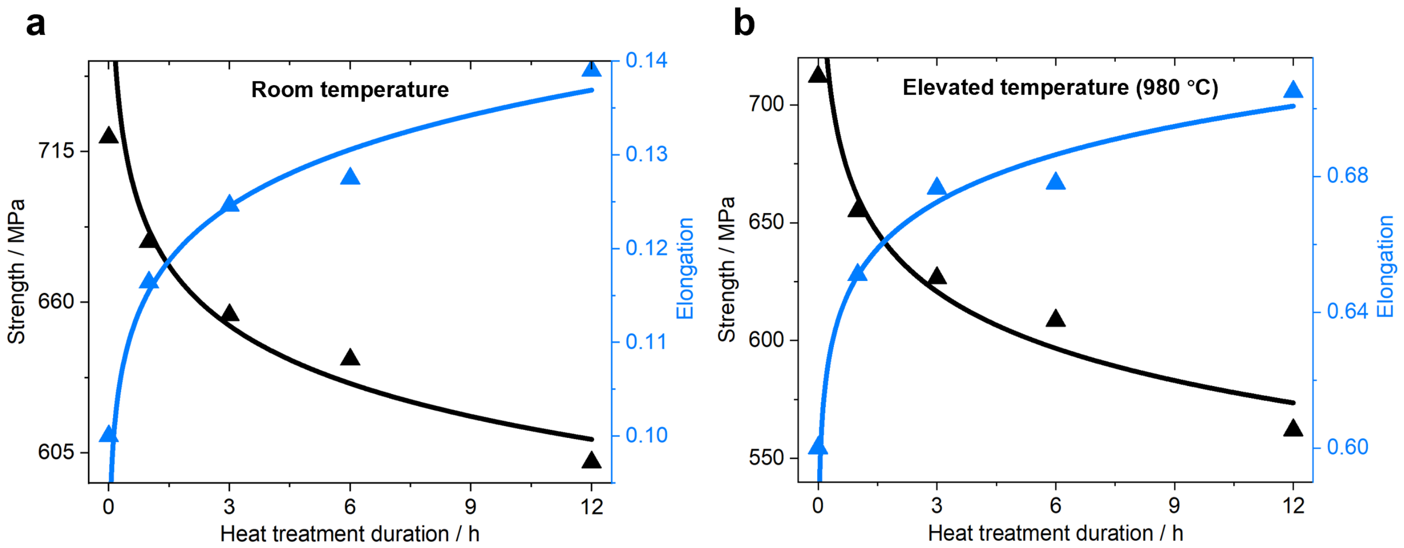

Fig. S19 Variations in strength and elongation along the trajectory of heat treatment in Fig.5 f and i: **(a-b)** under RT and 980 °C, respectively. The selected cases are representative, as they correspond to the lowest and highest investigated temperatures in this work. The scatters at varying heat treatment durations are extracted from the mapping between intensities of TGMs and mechanical properties in **Fig.5 f** and **i**, with the curves fitted based on the power law to explicitly express the strength and elongation using heat treatment durations. Based on the explicit correlations in **a** and **b**, the inverse-tailoring approach has been established, in which the heat treatment duration can be selected for the expected combination of strength and elongation at both RT and 980 °C.

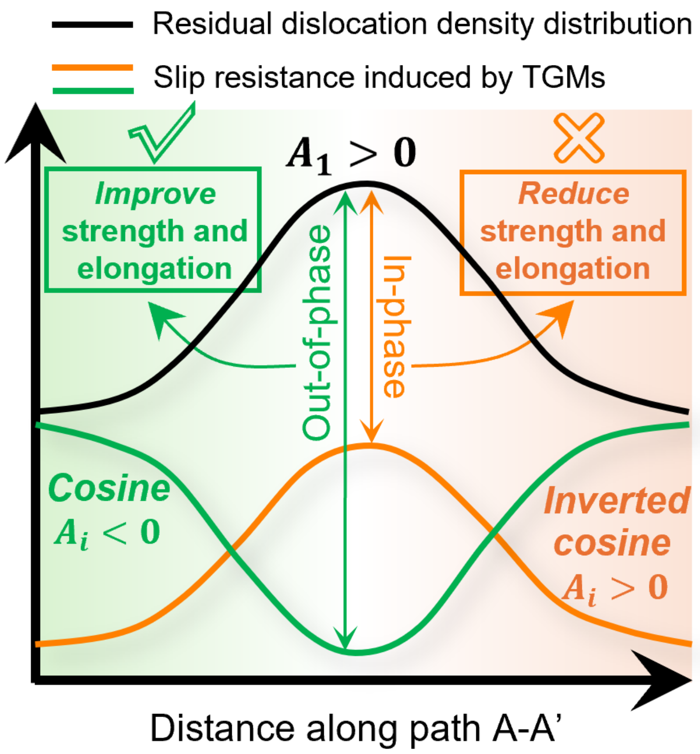

Fig. S20 Schematic diagram illustrating the general role of TGMs’ impacts on the mechanical properties of AM SXs: The path A-A’ is labeled in **Fig. 4 a**.

Supplementary Methods 1: details of crystal plasticity modeling

The total deformation gradient $\mathbf{F}$ can be decomposed into elastic part $\mathbf{F}_\mathrm{e}$ and plastic part $\mathbf{F}_\mathrm{p}$ [21,22]:

$$\mathbf{F} = \mathbf{F}_\mathrm{e}\mathbf{F}_\mathrm{p} \tag{S.1}$$

where the $\mathbf{F}_{\mathrm{p},0}$ is incorporated as the initial $\mathbf{F}_\mathrm{p}$ in simulation, to consider the residual deformation and residual stresses induced by the AM process.

The Green–Lagrange elastic strain tensor is written as[23]:

$$\boldsymbol{\varepsilon}_\mathrm{e} = \frac{1}{2}(\mathbf{F}_\mathrm{e}{}^\mathrm{T}\mathbf{F}_\mathrm{e} - \mathbf{I}) \tag{S.2}$$

where $\mathbf{I}$ is the identity matrix. Afterwards, the 2nd Piola–Kirchhoff stress tensor can be calculated as[24]:

$$\boldsymbol{\sigma} = \mathbf{C} : \boldsymbol{\varepsilon}_\mathrm{e} \tag{S.3}$$

where $\mathbf{C}$ is the elasticity constant tensor in the global coordinate system.

The shear strain rates $\dot{\gamma}^s$ across all slip systems collectively contribute to the plastic strain rate[25]:

$$\dot{\boldsymbol{\varepsilon}}_\mathrm{p} = \dot{\mathbf{F}}_\mathrm{p}\mathbf{F}_\mathrm{p}{}^{-1} = \sum_{s=1}^{N_\mathrm{slip}}(\mathbf{m}^s \otimes \mathbf{n}^s)\dot{\gamma}^s \tag{S.4}$$

where N_slip is the number of slip systems, $\mathbf{m}^s$ is the unit vector normal to the slip plane, and $\mathbf{n}^s$ is the unit vector along the slip direction. For the Ni-based SX alloys with FCC lattice, the slip systems are {111}<110>.

For the Eq. (3) in **3.4 Section**, the τ_c^s can be decomposed into[20,26]:

$$\begin{aligned} \tau_{\mathrm{c},1}^s &= \tau_0^s + \tau_\mathrm{solution}^s + \tau_\mathrm{dislocation}^s + \tau_\mathrm{Orowan}^s && \text{outside SB} \\ \tau_{\mathrm{c},1}^s &= \tau_0^s + \tau_\mathrm{solution}^s + \tau_\mathrm{dislocation}^s + (1-r)\tau_\mathrm{Orowan}^s && \text{inside SB} \end{aligned} \tag{S.5}$$

where τ_0^s, τ_solution^s, $\tau_\mathrm{dislocation}^s$, and τ_Orowan^s represent the slip resistances contributed by lattice friction, solid-solution strengthening, dislocation hardening, and dislocation bypassing of γ’,

respectively. The plasticity of γ' phases is neglected, as the plastic deformation is mainly contributed by the γ matrix[27-29]. The variable r represents the ratio of dislocation shearing of γ′, which linearly evolves with applied strain. When all γ′ phases are sheared by dislocations (r=1), the dislocations no longer bypass γ′ via the Orowan mechanism, resulting in the absence of τ^{s}_{Orowan}, thus leading to strain localization within slip band.

To simulate the slip band induced by dislocation shearing γ' precipitates, the criterion for slip band initiation and propagation of slip band boundaries is given as[12]:

$$\tau^{s}_{\text{shear}} = \frac{\gamma_{\text{APB}}}{2b}\left[\left(\frac{12\gamma_{\text{APB}} f_{\text{p}} d_{\text{p}}}{\pi G b^{2}}\right)^{1/2} - f_{\text{p}}\right] \tag{S.6}$$

where τ^{s}_{shear} is the critical resolved shear stress for the dislocation shearing of γ', G is the shear modulus, b is the norm of the Burgers vector, γ_{APB} is the anti-phase boundary (APB) energy, and f_{p} and d_{p} are the local fraction and average size of γ' precipitates.

The $\tau^{s}_{\text{solution}}$ in Eq.(S.5) is used to capture the solid-solution strengthening effect within γ matrix, which is written as[30]:

$$\tau^{s}_{\text{solution}} = \left(\sum_{i=1}^{n_{\text{s}}} k_i^2 c_i\right)^{1/2} \tag{S.7}$$

where c_i are the atomic percentages of solid-solution elements within the matrix channel, n_s is the number of solid-solution elements, and k_i are constants that can be found in the reference[30]. The EPMA results characterizing the local average concentration shown in **Fig. S9 a**, are utilized to determine the distribution of c_i in Eq.(S.7). It should be noted that Al and Ti are not involved in $\tau^{s}_{\text{solution}}$, since they mainly contribute to the formation of γ' phases, with marginal distribution within γ matrix[12]. The c_i for the involved solid-solution elements is converted into the concentration within γ matrix.

The $\tau^{s}_{\text{dislocation}}$ in Eq.(S.5) can be expressed by GND density and immobile statistically stored dislocation (SSD) density[31]:

$$\tau_{\text{dislocation}}^{s} = \varphi_0 G b \sqrt{\sum_{\alpha=1}^{N_{\text{slip}}} \chi_{\alpha}^{s} \left(\left|\rho_{\text{SSD}}^{\alpha}\right| + \left|\rho_{\text{GNDe}}^{\alpha}\right| + \left|\rho_{\text{GNDs}}^{\alpha}\right| \right)} \tag{S.8}$$

where $\rho_{\text{GNDe}}^{\alpha}$ and $\rho_{\text{GNDs}}^{\alpha}$ are the edge and screw components of structure-induced GND density, $\rho_{\text{SSD}}^{\alpha}$ is the immobile SSD density, φ_0 is the pre-factor of Taylor hardening law, and χ_{α}^{s} is the latent hardening matrix capturing the hardening interactions between different slip systems. The evolution of immobile SSD can be expressed as[32]:

$$\dot{\rho}_{\text{SSD}}^{s} = (K\sqrt{\left|\rho_{\text{SSD}}^{s}\right| + \left|\rho_{\text{GNDe}}^{s}\right| + \left|\rho_{\text{GNDs}}^{s}\right|} - 2y_c\left|\rho_{\text{SSD}}^{s}\right|)\frac{|\dot{\gamma}^{s}|}{b} \tag{S.9}$$

where K is a coefficient representing the accumulation rate, and y_c is the critical annihilation distance for SSD.

The Orowan stress τ_{Orowan}^{s} in Eq.(S.5) captures the slip resistance contributed by dislocations bypassing of precipitates, which can be written as[33]:

$$\tau_{\text{Orowan}}^{s} = \frac{Gb}{w} \tag{S.10}$$

where w is the matrix channel width.

Before applying the external load on RVE, we have considered the pre-loading of residual deformation $\mathbf{F}_{\text{p},0}$ (Eq.(S.1)). The detailed procedure is shown in **Fig. S21 a**. Based on the analysis in **Fig. S6**, we only consider the initial distribution of $F_{\text{p},0}^{\text{xx}}$, which is a dominant component in $\mathbf{F}_{\text{p},0}$. The $F_{\text{p},0}^{\text{yy}}$, $F_{\text{p},0}^{\text{zz}}$ are given as 1, and the other components in $\mathbf{F}_{\text{p},0}$ are set to 0. As the TEM observation (**Fig 1 b**) shows fewer dislocations at the dendrite core, the trough value of $F_{\text{p},0}^{\text{xx}}$ at the dendrite core is given as 1. The peak value of $F_{\text{p},0}^{\text{xx}}$ at inter-dendrite is calibrated by the DIC mapping of lateral strain (**Fig 3 b2**), with calibration results shown in **Fig 6 g**.

Regarding the specific case of SXs under uniaxial tension along [001], we develop a method to determine the AM-induced initial SSDs at individual slip systems. First, the residual deformation $\mathbf{F}_{\text{p},0}$ is simplified as uniaxial strain, based on the small deformation assumption[34]:

$$\varepsilon_{p,0}^{xx} = F_{p,0}^{xx} - 1 \quad \text{(S.11)}$$

As illustrated in **Fig. S21 b**, under the uniaxial tension and plastic strain along [001] orientation, there are 4 zero components and 8 non-zero components for the resolved shear strains, as the Schmid factors for the orange components equal 0. Meanwhile, since the 8 non-zero components share the same Schmid factor, these 8 components have the same absolute value of $\bar{\gamma}$. Based on Eq.(S.4), $\varepsilon_{p,0}^{xx}$ can be directly expressed by $\bar{\gamma}$ using a linear relationship, without any unsolved variables. Then, we can solve $\bar{\gamma}$ for the slip systems with non-zero resolved shear strains. Finally, the SSD densities at individual slip systems can be solved based on the finite difference scheme of Eq.(S.9).

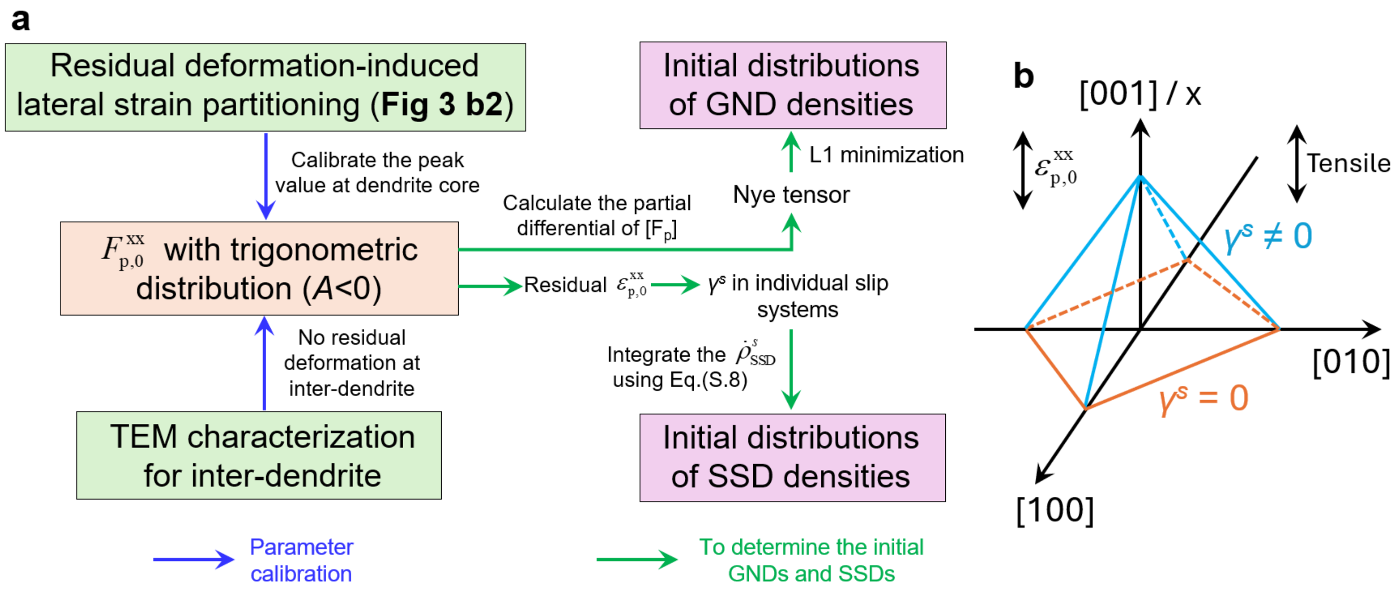

Fig. S21 Procedure to determine AM-induced residual deformation and initial SSDs/GNDs in CPFE simulation: (a) The flowchart to calibrate the initial $\mathbf{F}_p$ and determine the initial GNDs and SSDs densities at individual slip systems. **(b)** Schematic diagram for all the slip systems in FCC, in which the zero and non-zero γ^s are marked in orange and blue, respectively.

Moreover, to determine the AM-induced initial GND densities at individual slip systems, the $\mathbf{F}_{p,0}$ is employed to calculate the Nye tensor $\boldsymbol{\alpha}$:

$$\boldsymbol{\alpha} = (\nabla \times \mathbf{F}_{p,0})^{T} \quad \text{(S.12)}$$

Then, the Nye tensor can be linked with the GND densities across slip systems[34]:

$$\boldsymbol{\alpha} = \mathbf{A} \cdot \boldsymbol{\rho}_{\mathbf{GND}} \tag{S.13}$$

where $\mathbf{A}$ is a crystal orientation-dependent matrix with a dimension of 9×18. In Eq.(S.13), we can establish 9 constraints, but there are 18 components of GND density for FCC[35]. To solve this, the L1 minimization algorithm is adopted to estimate the GNDs at individual slip systems in each element [36]. Based on the above approach, the maximum initial GND density is estimated to be less than 1 μm^{-2}, which is two orders of magnitude lower than the initial SSD density. To save computational cost, the initial GNDs are not considered.

Moreover, the simulations are conducted under the open-source MOOSE platform[37]. In the RVE shown in **Fig. 6 a**, there are 30, 30, and 60 elements within the gauge length along x, y, z directions. The solver is based on the preconditioned Jacobian-free Newton-Krylov method, which provides an increased computational efficiency[38].

Table S3 Calibrated main parameters used in CPFE simulation.

	Symbol	Value	Unit	Calibration method
Elastic properties	C_{11}<br>C_{12}<br>C_{44}	135<br>96<br>65	GPa	Fitting the experimental stress-strain curves within elastic regime
Flow rule	n	0.1	-	Fitting the stress-strain curves at RT
	$\dot{\gamma}_0$	5E-6	s^{-1}	
	τ_0^s	35 (<980 °C)<br>12 (≥980°C)	MPa	
Solid-solution strengthening	k_{Al}<br>k_{Cr}<br>k_{Co}<br>k_{Ti}	225<br>337<br>39.4<br>775	MPa	From reference[12,30]
Dislocation bypassing of precipitate	G	35	GPa	From elastic properties
	b	0.255	nm	Calculated using lattice constant[26]
Dislocation hardening	φ_0	0.10 (RT)<br>0.2 (760 °C)<br>0.14 (850 °C)<br>0.30 (900 °C)<br>0.10 (980 °C)	-	Fitting the stress-strain curves at different temperatures

	y_c	0 (≤760 °C)<br>0.02 (>760°C)	μm	
	K	0.35	-	
	χ_s^α	1 (α, s at same slip plane)<br>1.4 (α, s at different slip plane)	-	From reference[31]
Dislocation shearing of precipitate	γ_{APB}	0.048 (RT)<br>0.05 (760 °C)<br>0.052 (900 °C)	J	Determined using the critical stresses of SB initiation at varying temperatures in DIC tests
Residual stresses	$F_{\mathrm{p},0}^{xx}$	0.01	-	Calibrated using the lateral strain distribution in DIC analysis
Damage evolution	E_{cr}	400	MPa	Fitting the strain softening regime in stress-strain curves
	D_{cr}	0.4 (with slip bands)<br>0.8 (without slip bands)	-	Calibrated by the elongations at different temperatures

Supplementary References

1 Li, Y., Yu, Y.-F., Wang, Z.-B., Liang, X.-Y., Kan, W.-B. & Lin, F. Additive Manufacturing of Nickel-Based Superalloy Single Crystals with IN-738 Alloy. *Acta Metallurgica Sinica (English Letters)* **35**, 369-374 (2022). https://doi.org:10.1007/s40195-021-01320-3

2 Yu, Y., Li, Y., Lin, F. & Yan, W. A multi-grid Cellular Automaton model for simulating dendrite growth and its application in additive manufacturing. *Additive Manufacturing* **47**, 102284 (2021). https://doi.org:https://doi.org/10.1016/j.addma.2021.102284

3 Fort, A., Landi, E., Moretti, R., Carbone, P. & Moschitta, A. Damped Sine Wave Parameter Extraction: Application to QCM-D Signals for Accurate Measurements. *IEEE Transactions on Instrumentation and Measurement* (2025).

4 Guo, B. *et al.* Segregation-dislocation self-organized structures ductilize a work-hardened medium entropy alloy. *Nature Communications* **16**, 1475 (2025). https://doi.org:10.1038/s41467-025-56710-3

5 Wang, Y. M. *et al.* Additively manufactured hierarchical stainless steels with high strength and ductility. *Nature Materials* **17**, 63-71 (2018). https://doi.org:10.1038/nmat5021

6 Huang, M. *et al.* Effect of γ forming element additions on the homogenization behavior and formation of hierarchical microstructures in Ni-based superalloys. *Journal of Alloys and Compounds* **975**, 172929 (2024). https://doi.org:https://doi.org/10.1016/j.jallcom.2023.172929

7 Neumeier, S. *et al.* Advanced Polycrystalline γ′-Strengthened CoNiCr-Based Superalloys. *Metallurgical and Materials Transactions A* **55**, 1319-1337 (2024). https://doi.org:10.1007/s11661-024-07319-6

8 *ImageJ-Pro Plus (Trial version)*, <https://imagej.net/ij/index.html> (2025).

9 Caccuri, V., Desmorat, R. & Cormier, J. Tensorial nature of γ′-rafting evolution in nickel-based single crystal superalloys. *Acta Materialia* **158**, 138-154 (2018). https://doi.org:https://doi.org/10.1016/j.actamat.2018.07.033

10 Guo, Z., Song, Z., Huang, D. & Yan, X. Matrix Channel Width Evolution of Single Crystal Superalloy Under Creep and Thermal Mechanical Fatigue: Experimental and Modeling Investigations. *Metals and Materials International* **28**, 2972-2986 (2022). https://doi.org:10.1007/s12540-022-01195-8

11 Wang, R. *et al.* Microstructure characteristics of a René N5 Ni-based single-crystal superalloy prepared by laser-directed energy deposition. *Additive Manufacturing* **61**, 103363 (2023). https://doi.org:https://doi.org/10.1016/j.addma.2022.103363

12 Reed, R. C. *The superalloys: fundamentals and applications*. (Cambridge university press, 2008).

13 Niu, H., Liu, Z., Wang, H., Wu, H., Liu, Q. & Fan, G. Effects of hot isostatic pressing on the micron-scale residual stress of nickel-based single-crystal superalloys. *Journal of Materials Science & Technology* **221**, 102-116 (2025).

https://doi.org:https://doi.org/10.1016/j.jmst.2024.09.036
14 Wan, W., Xu, Y., Yu, X. & Dunne, F. P. E. Microstructurally-sensitive fatigue crack nucleation in a Zircaloy-4 alloy. *Journal of the Mechanics and Physics of Solids* **180**, 105417 (2023). https://doi.org:https://doi.org/10.1016/j.jmps.2023.105417
15 Gribbin, S., Bicknell, J., Jorgensen, L., Tsukrov, I. & Knezevic, M. Low cycle fatigue behavior of direct metal laser sintered Inconel alloy 718. *International Journal of Fatigue* **93**, 156-167 (2016). https://doi.org:https://doi.org/10.1016/j.ijfatigue.2016.08.019
16 Huichen Yu, X. W. *Materials Data Manual in Aircraft Engine Design (4th Edition).* (Chinese Aviation Industy Press 2010).
17 Qu, Z. *et al.* High fatigue resistance in a titanium alloy via near-void-free 3D printing. *Nature* **626**, 999-1004 (2024). https://doi.org:10.1038/s41586-024-07048-1
18 Stinville, J. C. *et al.* On the origins of fatigue strength in crystalline metallic materials. *Science* **377**, 1065-1071 (2022). https://doi.org:10.1126/science.abn0392
19 Sidharth, R., Stinville, J. C. & Sehitoglu, H. Fatigue and fracture of shape memory alloys in the nanoscale: An in-situ TEM study. *Scripta Materialia* **234**, 115577 (2023). https://doi.org:https://doi.org/10.1016/j.scriptamat.2023.115577
20 Guo, Z. *et al.* Slip Band Evolution Behavior near Circular Hole on Single Crystal Superalloy: Experiment and Simulation. *International Journal of Plasticity* **165**, 103600 (2023). https://doi.org:10.1016/j.ijplas.2023.103600
21 Kalidindi, S. R. Incorporation of deformation twinning in crystal plasticity models. *J. Mech. Phys. Solids* **46**, 267-290 (1998). https://doi.org:https://doi.org/10.1016/S0022-5096(97)00051-3
22 Vujošević, L. & Lubarda, V. Finite-strain thermoelasticity based on multiplicative decomposition of deformation gradient. *Theor. Appl. Mech.*, 379-399 (2002). https://doi.org:https://doi.org/10.2298/TAM0229379V
23 Pedersen, P. Axisymmetric analytical stiffness matrices with Green-Lagrange strains. *Comput. Mech.* **35**, 227-235 (2005). https://doi.org:https://doi.org/10.1007/s00466-004-0619-4
24 Addessio, F., Luscher, D., Cawkwell, M. & Ramos, K. A single-crystal model for the high-strain rate deformation of cyclotrimethylene trinitramine including phase transformations and plastic slip. *Journal of Applied Physics* **121** (2017).
25 Xu, Y., Wan, W. & Dunne, F. Microstructural fracture mechanics: Stored energy density at fatigue cracks. *J. Mech. Phys. Solids* **146** (2020). https://doi.org:https://doi.org/10.1016/j.jmps.2020.104209
26 Guo, Z. *et al.* A dislocation-based damage-coupled constitutive model for single crystal superalloy: Unveiling the effect of secondary orientation on creep life of circular hole. *International Journal of Plasticity* **173**, 103874 (2024). https://doi.org:10.1016/j.ijplas.2024.103874
27 Guo, Z., Huang, D. & Yan, X. Physics-based modeling of γ/γ′ microstructure evolution and creep constitutive relation for single crystal superalloy. *International Journal of Plasticity* **137**, 102916 (2021). https://doi.org:https://doi.org/10.1016/j.ijplas.2020.102916
28 Yang, M. *et al.* A phase-field model for creep behavior in nickel-base single-crystal superalloy: Coupled with creep damage. *Scripta Materialia* **147**, 16-20 (2018). https://doi.org:https://doi.org/10.1016/j.scriptamat.2017.12.008
29 Lu, G., Zhao, Y., Zhao, T., Chen, Y., Wang, W. Y. & Wen, Z. Hydrogen trapping and embrittlement in a second-generation Ni-based single crystal superalloy. *Materials Science and Engineering: A* **915**, 147188 (2024). https://doi.org:https://doi.org/10.1016/j.msea.2024.147188
30 Roth, H., Davis, C. & Thomson, R. Modeling solid solution strengthening in nickel alloys. *Metall. Mater. Trans. A* **28**, 1329-1335 (1997). https://doi.org:https://doi.org/10.1007/s11661-997-0268-2
31 Bayley, C. J., Brekelmans, W. A. M. & Geers, M. G. D. A comparison of dislocation induced back stress formulations in strain gradient crystal plasticity. *Int. J. Solids Struct.* **43**, 7268-7286 (2006). https://doi.org:https://doi.org/10.1016/j.ijsolstr.2006.05.011
32 Hu, D., Guo, Z., Grilli, N., Tay, A., Lu, Z. & Yan, W. Understanding the strain localization in additively manufactured materials: Micro-scale tensile tests and crystal plasticity modeling. *International Journal of Plasticity* **177**, 103981 (2024). https://doi.org:https://doi.org/10.1016/j.ijplas.2024.103981

33 le Graverend, J.-B. A hardening-based damage model for fast-evolving microstructures: Application to Ni-based single crystal superalloys. *Int. J. Plast.* **123**, 1-21 (2019). https://doi.org:https://doi.org/10.1016/j.ijplas.2019.03.012
34 Das, S., Hofmann, F. & Tarleton, E. Consistent determination of geometrically necessary dislocation density from simulations and experiments. *International Journal of Plasticity* **109**, 18-42 (2018). https://doi.org:https://doi.org/10.1016/j.ijplas.2018.05.001
35 Zhong, H. *et al.* Resolving localized geometrically necessary dislocation densities in Al-Mg polycrystal via in situ EBSD. *Acta Materialia* **279**, 120290 (2024). https://doi.org:https://doi.org/10.1016/j.actamat.2024.120290
36 Arsenlis, A. & Parks, D. M. Crystallographic aspects of geometrically-necessary and statistically-stored dislocation density. *Acta Materialia* **47**, 1597-1611 (1999). https://doi.org:10.1016/S1359-6454(99)00020-8
37 Permann, C. J. *et al.* MOOSE: Enabling massively parallel multiphysics simulation. *SoftwareX* **11**, 100430 (2020). https://doi.org:https://doi.org/10.1016/j.softx.2020.100430
38 Chockalingam, K., Tonks, M. R., Hales, J. D., Gaston, D. R., Millett, P. C. & Zhang, L. Crystal plasticity with Jacobian-Free Newton–Krylov. *Computational Mechanics* **51**, 617-627 (2013). https://doi.org:10.1007/s00466-012-0741-7
