# Supplementary material for: Trigonometric gradient microstructures in additively manufactured single crystals enable strength-ductility synergy and programmable performance
Source: Nat Commun. 2025 Nov 11;16:9936. doi: 10.1038/s41467-025-64874-1 (PMC12606096; doi:10.1038/s41467-025-64874-1)
Supplement: Supplementary file 2 — Description of Additional Supplementary Files [file 41467_2025_64874_MOESM2_ESM.pdf]

Description of Additional Supplementary Files

File Name: Supplementary Movie 1

Description: CPFE simulation results for dendrite-scale deformation at room temperature

File Name: Supplementary Movie 2

Description: CPFE simulation results for dendrite-scale deformation at 980°C
